# Efficacy of novel antibacterial compounds targeting histidine kinase YycG protein

**DOI:** 10.1007/s00253-014-5685-8

**Published:** 2014-04-17

**Authors:** Huayong Liu, Dan Zhao, Jun Chang, Liang Yan, Fuju Zhao, Youcong Wu, Tao Xu, Ting Gong, Li Chen, Nianan He, Yang Wu, Shiqing Han, Di Qu

**Affiliations:** 1 Key Laboratory of Medical Molecular Virology of Ministries of Education and Health, School of Basic Medical Science and Institutes of Biomedical Sciences, Shanghai Medical College of Fudan University, Shanghai, 200032 China; 2 College of Biotechnology and Pharmaceutical Engineering, Nanjing University of Technology, Nanjing, 210009 China; 3 Department of Clinical Laboratory, Huadong Hospital, Fudan University, Shanghai, China; 4 Department of Natural Products Chemistry, School of Pharmacy, Fudan University, Shanghai, China; 5 Department of Ultrasound, Anhui Provincial Hospital of Anhui Medical University, Hefei, 230001 China

**Keywords:** *Staphylococcus epidermidis*, Methicillin-resistant *Staphylococcus aureus* (MRSA), Antibacterial, Minimal inhibitory concentration (MIC), Minimal bactericidal concentration (MBC), Antibiofilm activity

## Abstract

Treating staphylococcal biofilm-associated infections is challenging. Based on the findings that compound 2 targeting the HK domain of *Staphylococcus epidermidis* YycG has bactericidal and antibiofilm activities against staphylococci, six newly synthesized derivatives were evaluated for their antibacterial activities. The six derivatives of compound 2 inhibited autophosphorylation of recombinant YycG′ and the IC_50_ values ranged from 24.2 to 71.2 μM. The derivatives displayed bactericidal activity against planktonic *S. epidermidis* or *Staphylococcus aureus* strains in the MIC range of 1.5–3.1 μM. All the derivatives had antibiofilm activities against the 6- and 24-h biofilms of *S. epidermidis*. Compared to the prototype compound 2, they had less cytotoxicity for Vero cells and less hemolytic activity for human erythrocytes. The derivatives showed antibacterial activities against clinical methicillin-resistant staphylococcal isolates. The structural modification of YycG inhibitors will assist the discovery of novel agents to eliminate biofilm infections and multidrug-resistant staphylococcal infections.

**Electronic supplementary material:**

The online version of this article (doi:10.1007/s00253-014-5685-8) contains supplementary material, which is available to authorized users.

## Introduction

*Staphylococcus epidermidis* and *Staphylococcus aureus* are common pathogens in medical device biofilm-associated infections (Knobloch et al. 2001; Yarwood et al. 2004). Their ability to attach onto biomaterial surfaces of implanted medical devices or to fragments of dead tissue and form biofilms results in chronic and refractory infections (Otto 2012b) that are resistant to antibiotics and to host defense clearance mechanisms (Spoering and Lewis 2001). *S. epidermidis* is a common cause of biofilm-associated infections, even though it is less virulent than *S. aureus* (Giacometti et al. 2000; O’Gara and Humphreys 2001). Biofilm-associated infections persist until the implanted medical device is removed, resulting in extra trauma and cost (Donlan and Costerton 2002; Kiran et al. 2010). Up to 25 % of orthopedic implants are subject to revision surgery due to biofilm infections (Mah and O’Toole 2001; Otto 2012a).

Currently available antibiotics for the treatment of bacterial infections are targeted at the planktonic cells, not the sessile cells in biofilms (Falsetta et al. 2012). Consequently, strategies against staphylococcal biofilm infections include targeting the systems regulating biofilm formation, such as two-component systems or quorum sensing systems; degrading the matrix to disperse the bacteria; developing a new generation of antibiotics; and adopting novel combinations of antimicrobial agents (West and Stock 2001; Yarwood et al. 2004). However, the mechanisms of multiple antibiotic resistance in *S. epidermidis* and *S. aureus* biofilms are complex. The biofilm matrix may decrease antibiotic diffusion into the biofilm structure, causing the bacteria to have less exposure to the antimicrobial compounds. Nutrient or oxygen depletion within the biofilm causes the cells to have low metabolic activity and a reduced growth rate, thus rendering biofilm bacteria resistant to antibiotics (Aendekerk et al. 2005; Walters et al. 2003). Most existing antibiotics fail to adequately penetrate the biofilm or have limited activity against surface-attached cells and cells with low metabolic activity (Kiedrowski and Horswill 2011). Vancomycin is regarded as an antibiotic of last resort against methicillin-resistant *S. aureus* (MRSA), methicillin-resistant *S. epidermidis* (MRSE), and other multiple antibiotic-resistant infections caused by gram-positive bacteria, but it has no significant effect on the bacteria in a biofilm (Qin et al. 2006; Roper et al. 2000). Daptomycin and linezolid are now available for biofilm-associated infections caused by staphylococci, but neither was found to be bactericidal against biofilm-embedded bacteria (Parra-Ruiz et al. 2012). More novel drugs are urgently required to combat staphylococcal biofilm-associated infections and the targeting of a bacterial two-component system (TCS) is the approach taken here.

A TCS, composed of a histidine kinase (HK) and a response regulator (RR), serves as a basic stimulus-response coupling mechanism by which bacteria sense and respond to environmental changes. TCSs have been found in bacteria, fungi, and plants, but not in vertebrates (Barrett and Hoch 1998; West and Stock 2001). YycFG is an essential TCS that is highly conserved in gram-positive bacteria with a low G + C content (Dubrac et al. 2007). It plays important roles in the growth, cell wall metabolism, and biofilm formation of pathogenic staphylococcal species (Winkler and Hoch 2008). It has been suggested that YycG or YycF may serve as potential targets for the development of novel antimicrobial agents (Fukushima et al. 2011; Szurmant et al. 2005; Turck and Bierbaum 2012).

We have previously described two YycG inhibitors that target the HK domain of *S. epidermidis* YycG and show bactericidal and antibiofilm activities against *S. epidermidis* and *S. aureus*. One of the two leading compounds is compound 2: {2-{4-{3-(2-ethylphenyl)-2-[(2-ethylphenyl)imino]-4-oxothiazolidin-5-ylidene}methyl}-2-methoxyphenoxy}acetic acid (Huang et al. 2012; Qin et al. 2006). To enhance the antimicrobial activities of compound 2 and reduce the toxicity to mammalian cells, the structure was optimized in a series of derivatives by substituting different functional groups (fluorine group, thiophene ring, etc.) while keeping the core structure intact (Dan Zhao et al. 2013). Six out of 56 newly synthesized derivatives of compound 2 were selected for their anti-*Staphylococcus* activity. In this study, we evaluated the antimicrobial activities of the six derivatives, including in vitro minimal inhibitory concentration (MIC), bactericidal activity, antibiofilm efficacy, YycG phosphorylation-inhibiting activity, potential toxicity, and in vivo effectiveness in a rabbit subcutaneous *S. epidermidis* biofilm infection model.

## Materials and methods

### Ethics statement

All procedures performed on rabbits were conducted according to relevant national and international guidelines (the Regulations for the Administration of Affairs Concerning Experimental Animals, China) and were approved by the Institutional Animal Care and Use Committee (IACUC) of Shanghai Medical College, Fudan University (IACUC Animal Project Number: 20110630).

### Bacterial strains, media, and derivatives of compound 2

**Fig. 1:**
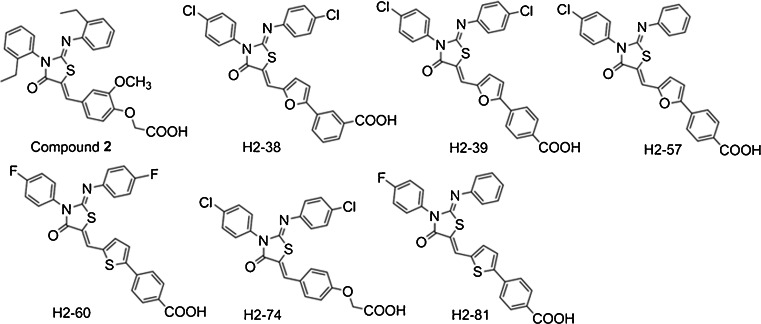
The structures of the thiazolidione derivatives. Based on the thiazolidione core structure of compound 2 {2-{4-{3-(2-ethylphenyl)-2-[(2-ethylphenyl)imino]-4-oxothiazolidin-5-ylidene}methyl}-2-methoxyphenoxy}acetic acid, six derivatives were designed and synthesized by modifying the functional groups through cyclization, aldol condensation, substitution and hydrolyzation reactions. H2-38, 3-{5-{{3-(4-chlorophenyl)-2-[(4-chlorophenyl)imino]-4-oxothiazolidin-5-ylidene}methyl}furan-2-yl}benzoic acid; H2-39, 4-{5-{{3-(4-chlorophenyl)-2-[(4-chlorophenyl)imino]-4-oxothiazolidin-5-ylidene}methyl}furan-2-yl}benzoic acid; H2-57, 4-{5-{{3-(4-chlorophenyl)-2-[(4-phenyl)imino]-4-oxothiazolidin-5-ylidene}methyl}furan-2-yl}benzoic acid; H2-60, 4-{5-{{3-(4-fluorophenyl)-2-[(4-fluorophenyl)imino]-4-oxothiazolidin-5-ylidene}methyl}thiophene-2-yl}benzoic acid; H2-74, 2-{4-{[3-(4-chlorophenyl)-2-[(4-chlorophenyl)imino]-4-oxothiazolidin-5-ylidene}methyl}phenoxy}acetic acid; H2-81, 4-{5-{{3-(4-fluorophenyl)-2-[(4-phenyl)imino]-4-oxothiazolidin-5-ylidene}methyl}thiophene-2-yl}benzoic acid

Bacterial strains *S. epidermidis* ATCC 12228 (nonbiofilm forming), *S. epidermidis* ATCC 35984 (biofilm forming), *S. aureus* ATCC 49230, and *S. aureus* ATCC 25923 were from the American Type Culture Collection (ATCC, Manassas, USA) and were cultured in tryptic soy broth medium (TSB; Oxoid Ltd., Basingstoke, England). Ten clinical methicillin-resistant staphylococcal isolates, five MRSA isolates and five MRSE isolates, were collected from Huadong Teaching Hospital in Shanghai. *Escherichia coli* ATCC 25922 was grown in Luria-Bertani (LB) broth. The six compounds used in this study (H2-38, H2-39, H2-57, H2-60, H2-74, and H2-81) were screened from 56 derivatives of compound 2 in which the functional groups were modified, but the thiazolidine core structure was unchanged. The compounds were synthesized by Nanjing University of Technology. To generate the derivative compounds, halogen atoms were introduced into 4-thiazolidinone compounds that contained carboxylic acid moieties of phenoxy-acetic acid, (5-vinyl-furan-2-yl)-benzoic acid or (5-vinyl-thiophene-2-yl)-benzoic acid. The derivatives were dissolved in dimethyl sulfoxide (DMSO; Amresco, USA) to 200 mM for use as stock solutions. The structures and systematic names of the derivatives are listed in Fig. 1.

### MIC and MBC assays

MICs of the derivatives for *S. epidermidis* and *S. aureus* were determined by the broth dilution method according to the National Committee for Clinical Laboratory (NCCLS) recommendations (Turnidge and Bordash 2007). Twofold dilutions of the derivatives in tubes containing 4 ml of Mueller-Hinton broth (MH, Sigma, Germany) were made to concentrations from 200 to 0.39 μM. Overnight cultures of the bacteria were adjusted to the 0.5 McFarland standards and inoculated 1:200 into the MH broth and then incubated at 37 °C for 16–20 h. The lowest concentration inhibiting visible growth of the bacteria was recorded as minimal inhibitory concentration (MIC). For the minimal bactericidal concentration (MBC) assay, a 100-μl aliquot from the MIC assay tubes with no visible turbidity was spread on freshly prepared MH agar plates and incubated at 37 °C for 24 h, and the colonies were counted. The MBC of the derivatives was determined as the lowest concentration of the derivative required to allow less than five colonies to grow on each plate (Qin et al. 2006).

### Inhibition of YycG′ autophosphorylation

Recombinant YycG (YycG′, amino acids 370–610) was prepared by expressing the HATPase_c and HisKA domains of YycG in *E. coli* BL21 (DE3). In brief, the sequence coding for YycG′ was amplified from the genome of *S. epidermidis* ATCC 35984 by PCR and cloned into the pET28a (+) vector, which inserts a GB1 tag (B1 domain of protein G, 56 residues) to make the recombinant fusion protein highly soluble and stable. The recombinant YycG′ protein was fused to the GB1 tag at its N-terminus end, expressed in *E. coli* BL21 (DE3) and purified by Ni^2+^ affinity chromatography on a Ni-NTA column (Qiagen, Germany). The purified YycG′ was cleaved by TEV protease and further purified by Ni-NTA and Superdex 75 gel filtration columns (GE Healthcare, USA).

The Kinase-Glo^™^ Luminescent Kinase Assay (Promega, Madison, WI, USA) was used to measure the inhibitory activities of the derivatives against the autophosphorylation activity of the recombinant YycG′ following the manufacturer’s recommendation. The derivatives were serially diluted from 200 to 1.6 μM with reaction buffer (40 mM Tris pH 8.0, 20 mM MgCl_2_, and 0.1 mg/ml BSA). Recombinant YycG′ (0.02 μg/μl) and diluted derivative were added into a 96-well plate and incubated at 25 °C for 30 min, and then 4 μM ATP was added into each well and incubated for another 30 min. Finally, 50 μl Kinase-Glo™ Reagent containing luciferase (kinase) and d-luciferin was added. The luminescent signal generated by luciferase was in proportion to the amount of ATP remaining in the reaction. Luminescence was detected by a Perkin Elmer Victor X5 luminescence plate reader, and the intensity was recorded as relative light units (RLU). The wells containing ATP and Kinase-Glo™ without YycG′ and derivatives or containing YycG′ plus ATP and Kinase-Glo™ without the derivatives were used as the controls. The percentage inhibition of protein phosphorylation (Rp) by the derivatives was calculated as follows:

$$ \mathrm{Rp}=\frac{\mathrm{RLU}\kern0.5em \left({{\mathrm{YycG}}^{'}+\mathrm{derivative}+\mathrm{ATP}+\mathrm{Kinase}-\mathrm{Glo}}^{\mathrm{TM}}\right)-\mathrm{RLU}\kern0.5em \left({{\mathrm{YycG}}^{'}+\mathrm{ATP}+\mathrm{Kinase}-\mathrm{Glo}}^{\mathrm{TM}}\right)}{\mathrm{RLU}\kern0.5em \left({\mathrm{ATP}+\mathrm{Kinase}-\mathrm{Glo}}^{\mathrm{TM}}\right)-\mathrm{RLU}\kern0.5em \left({{\mathrm{YycG}}^{'}+\mathrm{ATP}+\mathrm{Kinase}-\mathrm{Glo}}^{\mathrm{TM}}\right)}\times 100 $$

The concentration of a derivative required to inhibit half of the autophosphorylation of recombinant YycG′ (half maximal inhibitory concentration, IC_50_) was calculated with Origin 8.0 software (Origin Lab, Northampton, USA). Three independent assays were carried out, and each was performed in quadruplicate.

### Microtiter plate assay of *S. epidermidis* biofilms

The effects of the derivatives on immature (6-h-old) biofilms and mature (24-h-old) biofilms of *S. epidermidis* ATCC 35984 were detected by using a semiquantitative microtiter plate assay (Stepanovic et al. 2007). An overnight culture of bacteria was inoculated 1:200 into TSB medium containing 0.25 % glucose and statically incubated in a polystyrene 96-well plate (200 μl per well) at 37 °C for 6 or 24 h. The medium containing any nonadherent bacteria was then removed, and serial dilutions of derivatives in 200 μl fresh TSB were added to the wells and incubated for another 16 h at 37 °C. The wells were then washed gently three times with phosphate buffered saline (PBS), air-dried, fixed with methanol, and stained with 2 % (*w*/*v*) crystal violet. The staining was visually assessed and scanned at 570 nm using a 96-well plate spectrophotometer (DTX880, Beckman Coulter, USA), and the minimal biofilm eradication concentration (MBEC) was measured (Ceri et al. 1999).

### Observation of *S. epidermidis* biofilms by confocal laser scanning microscopy

The effects of the derivatives on bacterial viability in mature (24-h-old) biofilms of *S. epidermidis* ATCC 35984 were evaluated by a confocal laser scanning microscope (CLSM), with Live/Dead staining (BacLight, Molecular Probes, USA) for assessing bacterial viability (Qin et al. 2006). A diluted overnight culture of the bacteria was inoculated into cell culture glass bottom dishes (WPI, USA) and incubated at 37 °C for 24 h. After removal of the suspension cultures, the derivatives in fresh TSB at fourfold MIC concentration were added and incubated at 37 °C for 16 h. The biofilms were washed with PBS and stained with SYTO9 and propidium iodide (PI), both of which were used at a concentration of 1 μM. SYTO9-stained live cells and PI-stained cells dead in biofilms were visualized by a Leica TCS SP5 confocal laser scanning microscope with a ×63 1.4-NA oil immersion objective. Three-dimensional biofilm images were created by IMARIS 7.0.0 software (Bitplane).

### Observation of *S. epidermidis* biofilms by scanning electron microscopy

*S. epidermidis* ATCC 35984 was statically incubated for 6 h in polystyrene 96-well plates at 37 °C, then the diluted derivatives H2-74 and H2-81 were added at concentrations of 4 × MIC in fresh TSB medium and incubated for another 16 h at 37 °C. After the incubation, the wells were washed with PBS, fixed with glutaraldehyde (2.5 % in PBS) for 2 h, and rinsed with PBS. All samples were mounted on SEM sample stubs, sputtered with platinum, and observed under a field emission scanning electron microscope (JSM-6700 F, Japan).

### Kinetics of killing planktonic *S. epidermidis*

An overnight culture of *S. epidermidis* ATCC 35984 was inoculated into 20 ml of fresh MH broth at a 1:200 dilution (10^6^ CFU/ml). Concentrations of H2-74 or H2-81 from 0.38 μM (1/4 × MIC) to 6.3 μM (4 × MIC) were added and incubated at 37 °C with shaking at 200 rpm for 12 h. Every 2 h, 100 μl of the culture was diluted and spread on agar plates. Viable bacteria (CFU) were counted and the rate and extent of killing were determined by plotting CFU/ml against time (D’Arezzo et al. 2012; Lee et al. 2008). This experiment was independently repeated three times.

### Cytotoxicity and hemolytic activity of the derivatives

Vero 76 cells (African green monkey cells) were used to determine the cytotoxicity of the derivatives with the Cell Proliferation Kit I (MTT) (Roche, Indianapolis, USA). The Vero cells were grown in Dulbecco’s modified Eagle’s medium (DMEM), supplemented with 5 % fetal calf serum, 100 U/ml penicillin, 100 mg/ml streptomycin, and 2 mM l-glutamine. After culturing in 5 % CO_2_ at 37 °C for 48 h, the cells were harvested and dispensed into 96-well cell culture plates containing 5 × 10^4^ cells per well in 100 μl. The cells were co-incubated with serial twofold dilutions of derivatives from 200 to 6.25 μM (six different concentrations) for 24 h at 37 °C in 5 % CO_2_. Then, 10 μl of the MTT labeling reagent (final concentration 0.5 mg/ml) was added to each well and incubated for 4 h. The solubilization solution (100 μl per well) was added to dissolve the purple formazan salts generated in the viable cells, and the absorbance of each well was measured at 595 nm. Cells treated with the solvent (0.1 % DMSO) were used as a negative control and the cells cultured only with DMEM medium served as a blank control. The inhibitory rate was calculated as follows:

$$ \mathrm{Inhibitory}\kern0.5em \mathrm{rate}\%\kern0.5em =\kern0.5em \frac{\mathrm{Ocontrol}-\mathrm{ODtest}}{\mathrm{ODcontrol}}\kern0.5em \times 100 $$

CC_50_ was defined as the concentration of the derivatives that inhibited the Vero cell growth by 50 % and was calculated with the Origin v8.0 software (Origin Lab, Northampton, USA) (Qin et al. 2006).

The hemolytic activities of the derivatives on healthy human erythrocytes were also determined. Healthy human erythrocytes resuspended in normal saline (NS) at 5 % (*v*/*v*) were co-incubated with the derivatives at final concentrations of MIC, 4 × MIC, or 200 μM for 1 h at 37 °C in 96-well microtiter plates. The suspensions were centrifuged at 350 *g* for 10 min and the supernatant (100 μl) was transferred to new wells and measured at 570 nm on a spectrophotometer (Benchmark Microplate Reader; Bio-Rad, Hercules, CA, USA). The percentage of hemolysis was calculated by normalizing against the absorbance of erythrocytes treated with 1 % Triton X-100, which caused complete hemolysis; 0.1 % DMSO-treated cells were used as a control (Miyoshi et al. 1997). The experiment was performed in quadruplicate wells and repeated independently three times.

### Rabbit subcutaneous *S. epidermidis* biofilm infection model

To test the effectiveness in vivo of H2-74 and H2-81, 24-h biofilms of *S. epidermidis* grown on polyethylene disks were imbedded subcutaneously into the New Zealand White rabbits and treated with the derivatives for 72 h in a modification of the method used previously (He et al. 2011). Disks were cut from polyethylene 96-well plates (8 mm diameter, 1 mm thickness), sterilized with 75 % ethanol and placed in a Petri dish (100 mm diameter). An overnight culture of *S. epidermidis* ATCC 35984 (20 ml) was inoculated into the dish and incubated at 37 °C for 24 h. The disks covered with biofilms were then implanted subcutaneously in female New Zealand White rabbits (2.0–2.5 kg) that had been anesthetized with pentobarbital sodium (5 mg/kg i.v.). Four incisions (10 mm) were made on the back of the rabbit along the spine bilaterally after removal of fur, and the sub cutis was carefully dissected to form a 2-cm × 3-cm cavity. Two biofilm-covered polyethylene disks were implanted into each cavity, and to minimize the effect of between-animal variation, four different treatments were given locally to each rabbit.

The treatments were as follows: 1 ml of H2-74 (6.3 μM, 4 × MIC) or H2-81 (6.3 μM, 4 × MIC) dissolved in NS was injected into the cavity locally after the biofilm-covered disks were implanted and the incisions were sutured, and the same dosages were administered at 24 and 48 h. Vancomycin (128 mg/l) and 0.1 % DMSO were administered as the controls. Twenty-four hours after the last treatment, the rabbits were euthanized and the implants were taken out with sterile forceps. Biofilms were scraped from the disks, and the viable bacteria were determined by CFU counting, as previously described (He et al. 2011). All animals were housed and used in compliance with the guidelines of the Institute of Animal Care and Use Committee, and the protocol was approved by the committee.

### Statistical analysis

Data from the rabbit model of *S. epidermidis* biofilm infection were compared by one-way analysis of variance (ANOVA) and Bonferroni’s multiple comparison test using the Origin v8.0 software (Origin Lab, Northampton, USA). A *p* value less than 0.05 was considered statistically significant.

## Results

Six of our 56 synthesized derivatives of leading compound 2 (H2-38, H2-39, H2-57, H2-60, H2-74, and H2-81) had exhibited low MICs (≤3.1 μM) against *S. epidermidis* ATCC 35984 (Huang et al. 2012; Qin et al. 2006) and were selected for further investigation of antimicrobial and antibiofilm activities.

### Inhibition of YycG′ autophosphorylation

The inhibition effects of the six derivatives on the autophosphorylation activity of the purified recombinant YycG′ were detected by using the Kinase-Glo^™^ Luminescent Kinase Assay. The recombinant YycG′ hydrolyzed ATP for its autophosphorylation in the reaction; the phosphorylation of YycG′ was inhibited when treated with the derivatives and the IC_50_ values of each derivatives were calculated. At a concentration of 100 μM, H2-38, H2-39, H2-57, H2-60, H2-74, and H2-81 inhibited the enzymatic autophosphorylation of YycG′ (0.13 μM) by 61.8, 56.3, 59.7, 55.4, 53.9, and 79.2 %, respectively. The IC_50_ values of the six derivatives ranged from 24.2 to 71.2 μM and the IC_50_ of the prototype compound 2 was 47.9 μM as shown in Table 1.

**Table 1:** Biological activities of the six derivatives of compound 2

**Derivatives** ^**a**^	**Molecular weight**	**MIC** ^b^ μ**M**)	**MBC** (μ**M**)	**MIC**/**MBC**	**MBEC** ^**b**^ (μ**M**)	**IC**_**50**_^**c**^ μ**M**)	**CC**_**50**_^**d.**^(μ**M**)	**Hemolysis** (%)^**e**^	
								**At MIC**	**200** μ**M**
Compound 2	519	25	100.0	1/4	100.0	47.9 ± 5.3	50	2.31 ± 0.35	5.24 ± 0.21
H2-38	534.02	1.5	12.5	1/8	12.5	41.4 ± 3.4	>200	0.14 ± 0.03	0.28 ± 0.03
H2-39	534.02	1.5	12.5	1/8	12.5	59.8 ± 1.4	>200	0.12 ± 0.03	0.41 ± 0.05
H2-57	500.06	3.1	12.5	1/4	12.5	47.1 ± 1.5	>200	0.14 ± 0.03	0.16 ± 0.04
H2-60	518.06	1.5	12.5	1/8	12.5	63.7 ± 2.3	>200	0.52 ± 0.06	0.84 ± 0.03
H2-74	498.02	1.5	25.0	1/16	50.0	71.2 ± 4.9	>200	0.14 ± 0.03	0.42 ± 0.03
H2-81	500.07	1.5	6.3	1/4	6.3	24.2 ± 1.2	>200	0.06 ± 0.09	0.10 ± 0.03

^a^Stock solutions (200 mM) of the derivatives were prepared in DMSO, and 200 μM was the highest concentration used in this study

^b^MIC, MBC, and MBEC represent minimal inhibitory concentration, minimal bactericidal concentration, and minimal biofilm eradication concentration of the derivatives against *S. epidermidis* ATCC 35984

^c^IC_50_ represents half maximal inhibitory concentration of the derivatives, which is the concentration needed to inhibit half of the autophosphorylation of recombinant YycG′ determined by the Kinase-Glo^™^ Luminescent Kinase Assay kit

^d^CC_50_ represents the derivative concentration that produces 50 % cytotoxicity effects on Vero cells. At 200 μM, cytotoxicity of the derivatives on the Vero cells was less than 3 % determined by the MTT assay with the Cell Proliferation Kit

^e^Hemolytic activities of the derivatives at the MICs and 200 μM were detected on healthy human erythrocytes

### Antimicrobial activities of the derivatives

All six derivatives inhibited the growth of *S. epidermidis* ATCC 35984 in MH broth; the MIC values of the derivatives ranged from 1.6 to 3.1 μM, and the MBC ranged from 6.3 to 25 μM, while the MIC and MBC of compound 2 were much higher which were 25 and 100 μM, respectively (Table 1). The ratios of MIC/MBC values of most derivatives (H2-38, H2-39, H2-57, H2-60, and H2-81) ranged from 1/2 to 1/8, whereas for H2-74, the ratio reached 1/16.

**Fig. 2:**
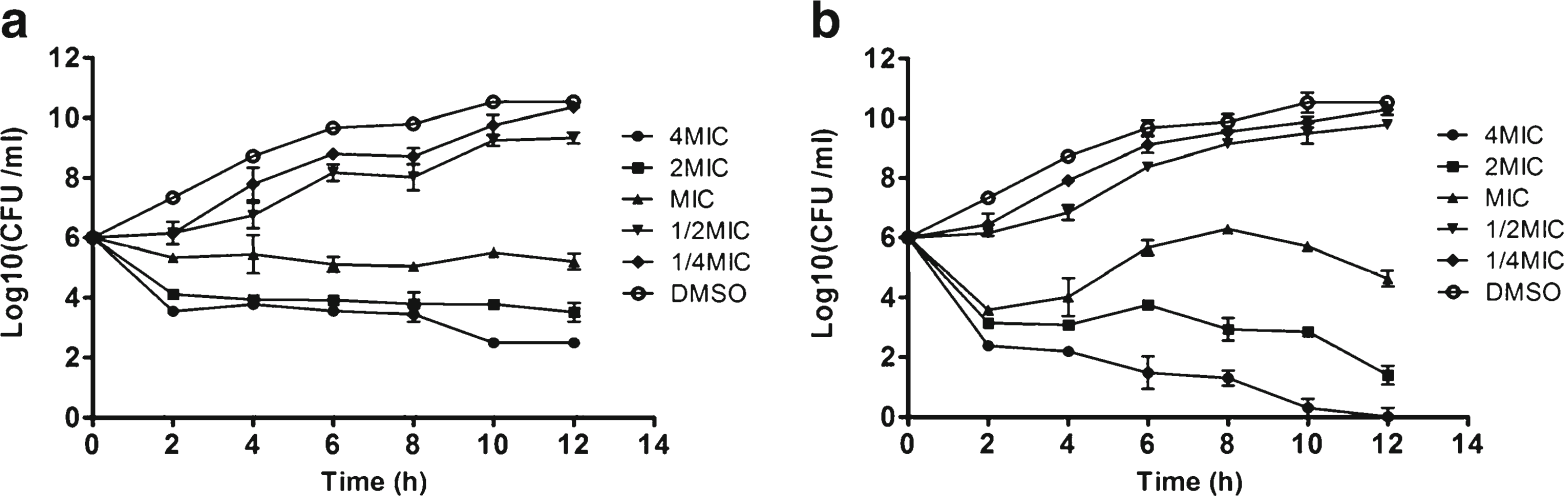
The kinetics of H2-74 and H2-81 action against *S. epidermidis* ATCC 35984. The bacteria were cultured with H2-74 (**a**) or H2-81 (**b**) at 1/4 × MIC, 1/2 × MIC, 1 × MIC, 2 × MIC, and 4 × MIC in 20 ml of fresh MH broth, at 37 °C for 12 h. Every 2 h, viable bacteria (CFU) were counted. The bacterial growth inhibition/killing was determined by plotting viable colony counts (CFU/ml) against time. The results are from three separate experiments and presented as the mean ± SD of triplicate samples

According to the different bacteriostatic and bactericidal activities of the derivatives, we chose H2-74 and H2-81 for further assay and tested killing kinetics against planktonic bacteria. In the initial treatment phase (from 2 to 6 h), at the concentrations of 1/4MIC (0.38 μM) and 1/2MIC (0.75 μM), H2-74 and H2-81 inhibited the growth of *S. epidermidis*, as shown in Fig. 2. When treated with H2-74 at 2 × MIC (3.1 μM for 12 h, the viable cells were decreased to 3.2 × 10^3^ CFU/ml (Fig. 2a), while with H2-81 treatment at 2 × MIC, the viability was decreased to less than 3 × 10^2^ CFU/ml (Fig. 2b). When treated with H2-81 at the concentration of 4 × MIC (6.3 μM) for 10 h, the viable cells decreased to fewer than 10 CFU/ml, but for H2-74, more than 3.1 × 10^2^ CFU/ml were left.

We further tested the antimicrobial activity of the six derivatives on two *S. aureus* standard strains (*S. aureus* ATCC 49230 and *S. aureus* ATCC25923) and ten clinical methicillin-resistant staphylococcal strains (five MRSE and five MRSA isolates). All six derivatives were able to inhibit the growth of *S. aureus* in addition to *S. epidermidis*, and the MIC values ranged from 1.6 to 6.3 μM (Table 2). They had no effect on the growth of *E. coli* strain ATCC 25922 at 200 μM, which is the highest concentration used in the present study.

**Table 2:** Anti-*Staphylococcus* activities of the derivatives

**Derivatives**	**MIC** ^**a**^ (μ**M**)					
	***S. epidermidis*** **ATCC 12228**	***S. aureus*** **ATCC 49230**	***S. aureus*** **ATCC 25923**	**Clinical MRSE strains** (***n*** = **5**)	**Clinical MRSA strains** (***n*** = **5**)	***E. coli*** **ATCC 25922** ^b^
Compound 2^c^	25	50	50	25–50	25–50	>200
H2-38	1.5	1.5	1.5	1.5–3.1	1.5–3.1	>200
H2-39	1.5	1.5	1.5	1.5–3.1	1.5–3.1	>200
H2-57	3.1	1.5	3.1	1.5–3.1	1.5–3.1	>200
H2-60	1.5	1.5	1.5	1.5–3.1	1.5–3.1	>200
H2-74	1.5	1.5	3.1	3.1–6.3	3.1–6.3	>200
H2-81	1.5	1.5	1.5	1.5	1.5	>200

^a^MIC, which represents minimal inhibitory concentration of the derivatives, was determined by the broth dilution (in tubes) method according to the standards of CLSI of the USA

^b^The derivatives did not inhibit the growth of *E. coli* ATCC 25922, even at the highest concentration used in the experiment

^c^MIC values for compound 2 were determined in this study

### Effect of the derivatives on *S. epidermidis* biofilms

The activities of the six derivatives on the both immature and mature biofilms of *S. epidermidis* ATCC 35984 were evaluated, and the MBEC was determined. When 6-h-old (immature) biofilms on 96-well plates were treated for 12 h, the six derivatives all inhibited biofilm formation. The MBEC ranged from 6.3 to 50 μM, lower than that of compound 2; H2-81 had the lowest MBEC and H2-74 the highest (Table 2). When 24-h-old (mature) biofilms were treated, no difference was found between the OD_570_ values of the treated and untreated biofilms (data not shown).

**Fig. 3:**
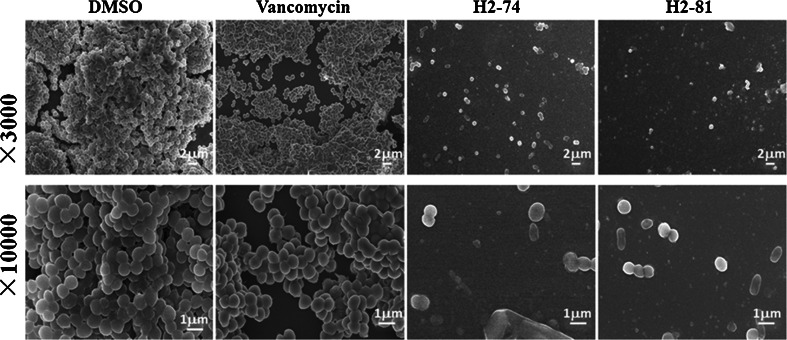
SEM of biofilms treated with the derivatives H2-74 or H2-81. An overnight culture of *S. epidermidis* ATCC 35984 was inoculated into polystyrene 96-well plates and incubated for 6 h. Then, DMSO, vancomycin (128 mg/l), or 4 × MIC of H2-74 and H2-81 was added into each well and incubated for 16 h. The biofilm samples were fixed with gluteraldehyde and observed by SEM

When 6-h-old biofilms on the polystyrene bottomed plates were treated with either H2-74 or H2-81 at a concentration of 4 × MIC, few bacteria on the plates were observed using scanning electron microscopy. When treated with vancomycin (128 mg/l), the morphology of the biofilms was similar to that treated with 0.1 % DMSO as a negative control (Fig. 3).

**Fig. 4:**
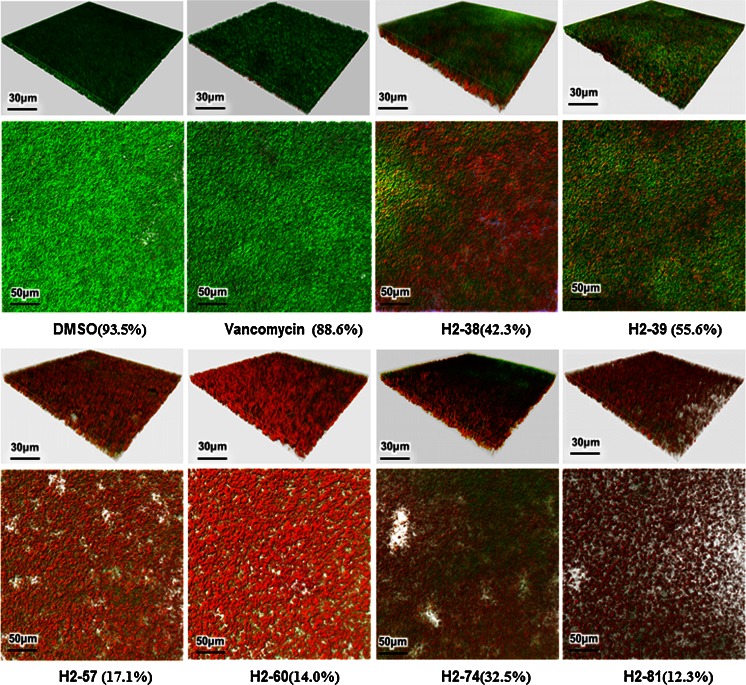
Bactericidal effect of derivatives on mature biofilms of *S. epidermidis* observed by CLSM. Overnight cultures of *S. epidermidis* ATCC 35984 were inoculated into FluoroDishes and incubated at 37 °C for 24 h, and then the biofilms were treated with derivatives at concentrations of 4 × MIC for another 16 h. The biofilms were visualized by CLSM with Live/Dead BacLight viability staining (SYTO9/PI). Cells stained with green fluorescence are viable and with red fluorescence are dead. The images of three-dimensional biofilm structure were created by IMARIS 7.0.0 software. The percentages of green bacteria in total bacteria were calculated by ImageJ using the intensity of green fluorescence divided by that of total fluorescence

When the bactericidal activity of the derivatives on mature (24-h-old) biofilms of *S. epidermidis* on glass was assessed by confocal microscopy with Live/Dead staining (CLSM), all six derivatives showed bactericidal activities at 4 × MIC. H2-81 reduced the viability to 12.3 %, while H2-60 and H2-57 reduced viability to 14 and 17.1 %, respectively. The proportion of viable cells in the biofilms treated with vancomycin (128 mg/l) was 88.6 %, similar to that of 0.1 % DMSO (Fig. 4).

### Cytotoxicity and hemolytic activity of the derivatives

When the derivatives at six different concentrations up to 200 μM were added onto Vero cells and incubated for 24 h at 37 °C, the formation of formazan in the MTT viability assay was inhibited by less than 3 %. The CC_50_ values of all the derivatives were therefore greater than 200 μM higher than that of the parent prototype compound (50 μM Cells treated with 0.1 % DMSO showed no cytotoxicity (Table 1).

At the MIC concentrations, all six derivatives lysed healthy human erythrocytes by less than 1 %, while the prototype compound 2 produced 2.31 ± 0.35 % hemolysis (Table 2). Even at the highest concentrations (200 μM), all of the derivatives gave no obvious hemolysis, whereas compound 2 gave 5.24 % hemolysis.

### Antimicrobial efficacy of the derivatives in a rabbit subcutaneous *S. epidermidis* biofilm infection model

**Fig. 5:**
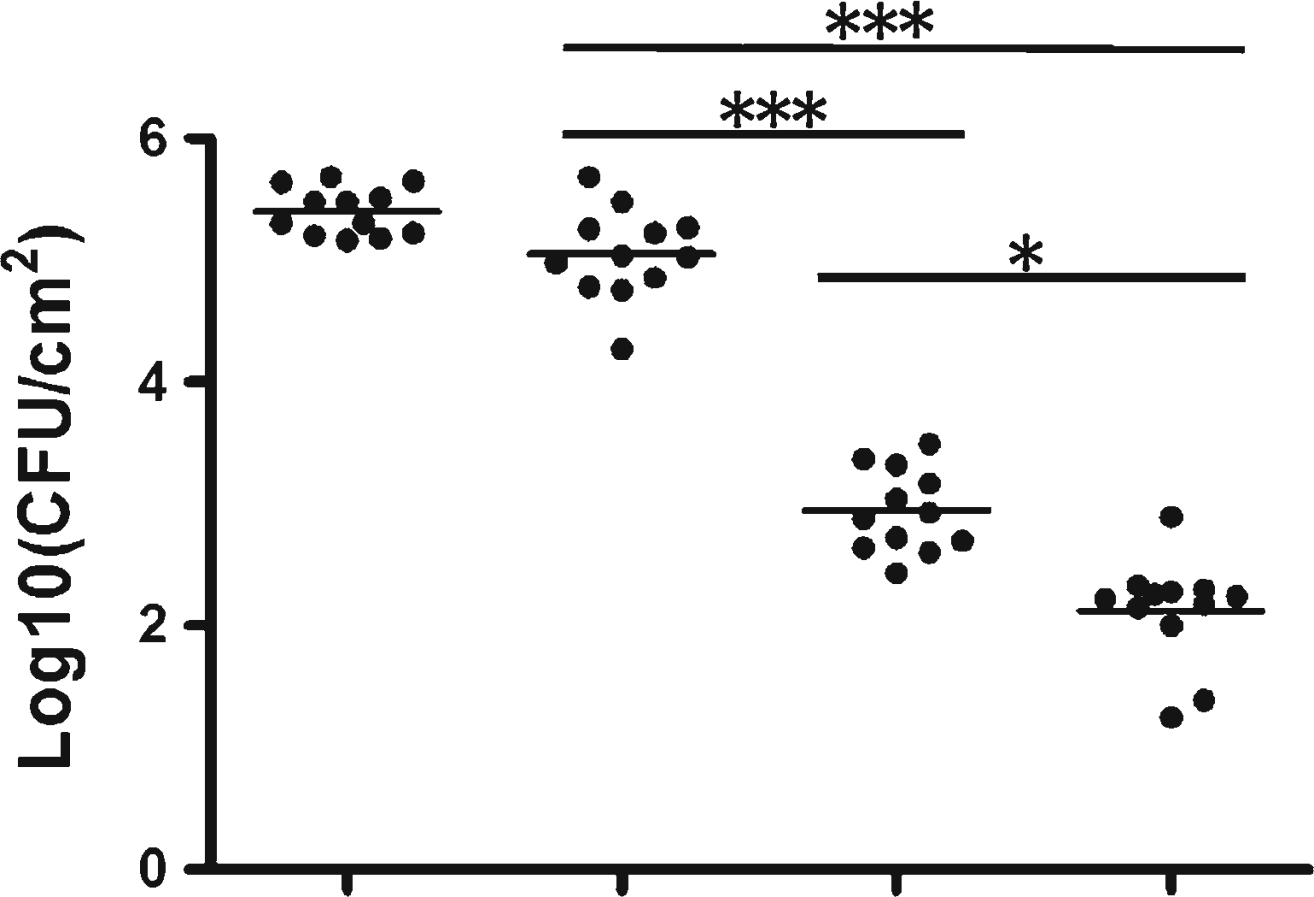
The viable bacteria recovered from the implanted disks in rabbit subcutaneous *S. epidermidis* infection model. The effects of H2-74 and H2-81 on 24-h biofilms of *S. epidermidis* ATCC 35984 were tested in a rabbit subcutaneous *S. epidermidis* infection model. Biofilm-covered polyethylene disks were implanted in subcutaneous cavities and exposed to H2-74 (6.3 μM, 4 × MIC) or H2-81 (6.3 μM, 4 × MIC) by three intra-cavity injections at 24-h intervals. Vancomycin (128 mg/l) and 0.1 % DMSO were administered as controls. Twenty-four hours after the last treatment, the implants were taken out and viable bacteria on the disks were determined by CFU counting. *Y*-axis represents the number of *S. epidermidis* CFU; *each dot* in the figure represents the count of viable cells from one disk. Data were analyzed by ANOVA with Bonferroni’s multiple comparison test; *p < 0.05 and ***p < 0.001 (*n* = 12)

We evaluated antibiofilm activities of H2-74 and H2-81 in vivo using a rabbit subcutaneous *S. epidermidis* biofilm infection model. When 24-h-old biofilms of *S. epidermidis* on polyethylene disks were implanted subcutaneously and exposed for 72 h to either H2-74 or H2-81 at 4 × MIC, bacterial viability was substantially reduced compared to DMSO or vancomycin controls (Fig. 5). CFUs in biofilms treated with H2-74 were reduced to 2.91 ± 0.42 log10 CFU/cm^2^ and H2-81 to 2.18 log10 CFU/cm^2^ (*p* < 0.05, *n* = 6), whereas the CFUs in the 0.1 % DMSO-treated biofilms were 5.3 ± 0.15 log10 CFU/cm^2^, and in the vancomycin-treated biofilms, 5.21 ± 0.24 log10 CFU/cm^2^.

## Discussion

Many new compounds with antibacterial activities have been discovered through structure-activity studies and library screening using the core scaffold of potential targets. These include inhibitors targeting the kinase activities of YycF and YycG (Watanabe et al. 2003). The YycFG TCS or its orthologs (also referred as WalRK) (Dubrac et al. 2007) have been found in most low G + C gram-positive bacteria, including many important pathogens. They play important roles in regulating bacterial murein and exopolysaccharide biosynthesis, biofilm formation, cell division, and virulence factor expression (Bisicchia et al. 2007; Dubrac and Msadek 2004; Ng et al. 2003; Winkler and Hoch 2008). Inhibitors against YycG or YycF are bactericidal for gram-positive pathogens (Okada et al. 2010). It has been reported that the inhibitors walkmycin B that targets YycG and walrycin A/B that targets YycF are effective against *Bacillus subtilis* and *S. aureus* at low MICs, though efficacy against biofilms has not been described (Okada et al. 2010; Watanabe et al. 2003).

In previous studies, we have found two leading compounds (compound 2 and compound 5) that target YycG and have antibiofilm activities against *S. epidermidis* (Qin et al. 2006). To improve the antibacterial activities of compound 2, we designed and synthesized a series of derivatives, keeping the thiazolidione core structure intact (Huang et al. 2012; Pan et al. 2010). Here, the anti-staphylococcal activities of 56 of the newly synthesized derivatives were tested, and six showed higher antibacterial activities (MIC ranged from 1.5 to 6.3 μM) than the leading compound 2 (MIC = 25 μM) and H2-28 (MIC = 3.1 μM) than previously reported (Huang et al. 2012). These six new derivatives exhibited antimicrobial activities against both MSRE and MSRA clinical isolates. All of them have halogen substituents (F or Cl) on phenyl rings of the thiazolidine core structure, indicating that introducing halogen elements maintained or improved the potent antibacterial activity of compound 2.

The six derivatives, especially H2-57, H2-74, and H2-81, were bactericidal against *S. epidermidis* cells in mature biofilms, and their antibiofilm activities have been improved compared to that of compound 2 and the derivatives studied previously (H2-10, H2-12, H2-20, H2-27, H2-28, H2-29) (Huang et al. 2012). At the highest concentration of the derivatives used in this study (200 μM), no cytotoxicity or hemolytic activities were observed. Furthermore, in the rabbit subcutaneous *S. epidermidis* biofilm infection model, the tested derivatives (H2-74 and H2-81) reduced bacterial viability, indicating their potential efficacy against clinical biofilm infection in vivo.

Although H2-74 and H2-81 showed the same bacteriostatic activities (MIC = 1.5 μM), they had different bactericidal efficacy; when *S. epidermidis* planktonic cells were treated with H2-81 at the concentration of 4 × MIC for 10 h only, ~10 CFU remained, while for H2-74, 3.2 × 10^2^ CFU were detected. Consistent with this, the IC_50_ of H2-74 (71.2 μM) was higher than that of H2-81 (24.2 μM). Comparison of the two derivatives’ structures shows that H2-74 contains a 4-phenoxy-acid fragment while H2-81 bears a 3-(5-thiophene-2-yl) benzoic acid fragment. The incorporation of a 2-phenylfuran moiety could enhance antibacterial activity of compounds (Ashok et al. 2007), and furan rings had been introduced into the derivatives H2-38, H2-39, and H2-57, which also displayed potent antibacterial activities and inhibitory effect on the phosphorylation of the YycG′ protein. The furan moiety was replaced with thiophene based on bioisosterism in H2-60 and H2-81, and both structures showed similar antibacterial activity and inhibitory effect on the phosphorylation of the YycG′ protein. This indicates that incorporation of a furan or thiophene ring and introducing halogen elements in the appropriate position may improve the antibacterial activities of the derivatives.

Combination antimicrobial therapy is widely used to take advantage of different mechanisms of action. It may potentiate the effect of individual antimicrobial agents by synergic action (Bijnsdorp et al. 2011; Cokol et al. 2011). The glycopeptide antibiotics, such as vancomycin, can bind the peptidoglycan side chains in the cell wall and prevent cross-linking during cell wall synthesis. Vancomycin has been considered to be one of the most reliable therapeutic agents against staphylococcal infections, but it is unable to clear biofilms even at high concentration (128 mg/l) (Climo et al. 1999; Darouiche et al. 1994; Tenover et al. 2001). Cefazolin has been used to treat various bacterial infections worldwide since the 1970s, and it can competitively inhibit the transpeptidases known as penicillin-binding proteins (PBPs) in the final transpeptidation step of the synthesis of peptidoglycan. YycGF also plays an important role in the synthesis of peptidoglycan in *B. subtilis* and *S. aureus* (Bisicchia et al. 2007; Dubrac and Msadek 2004). In a preliminary study, we assessed the effects of the combined application of the derivatives with vancomycin and cefazolin against *S. epidermidis* ATCC 35984 according to the method of Odds (2003) (Cottagnoud et al. 2003). A synergistic effect on *S. epidermidis* was observed when H2-81 was combined with vancomycin or cefazolin. No antagonism was observed between the derivatives and vancomycin or cefazolin (Table S1). These observations suggested that the derivatives may be used either alone or in association with other antibiotics. The potential synergistic effects in drug combinations warrant further investigation with clinical staphylococcal strains and multiresistant isolates.

Biofilms are highly resistant to clearance by most antimicrobial therapies because of the complicated multicellular architecture (Kittinger et al. 2011). The subcutaneous foreign body infection animal models are established for studying the effect of remedies on medical device-related biofilms, including mice, rat, and rabbit models (Coenye and Nelis 2010). Compared with other subcutaneous foreign body biofilm infection models, rabbit models are easy to manipulate and can be treated with different derivatives at the same time to reduce individual differences. In the present study, we used the rabbit subcutaneous *S. epidermis* biofilm infection models to evaluate the antibiofilm efficacy of the derivatives in vivo. After the treatment of H2-74 and H2-81 locally, CFUs in the biofilms were significantly reduced compared with untreated controls. This is in concordance with antibiofilm efficacy in vitro.

In summary, the bactericidal and antibiofilm activities of the six newly designed derivatives of YycG inhibitors are improved compared to prototype compound 2. However, the antimicrobial efficacy of derivatives should be further improved by additional modification of the compound structures. Studies of the toxicity in vivo and of the preclinical druggability of the derivatives will also be needed before there might be clinical application against biofilm-associated infections and multidrug-resistant bacterial infections.

## Electronic supplementary material

Below is the link to the electronic supplementary material.

ESM 1 (PDF 159 kb)
