# Supplementary material for: Efficacy of novel antibacterial compounds targeting histidine kinase YycG protein
Source: Appl Microbiol Biotechnol. 2014 Apr 17;98(13):6003–13. doi: 10.1007/s00253-014-5685-8 (PMC4057637; doi:10.1007/s00253-014-5685-8)
Supplement: Supplementary file 1 — (PDF 159 kb) [file 253_2014_5685_MOESM1_ESM.pdf]

Supplementary material

Applied Microbiology and Biotechnology (AMAB-D-13-02692)

Efficacy of novel antibacterial compounds targeting histidine kinase YycG protein

Huayong Liu[†], Dan Zhao[†], Jun Chang, Liang Yan , Fuju Zhao, Youcong Wu, Tao Xu, Ting Gong, Li Chen, Nianan He, Yang Wu, Shiqing Han*, and Di Qu*

[1] Key Laboratory of Medical Molecular Virology of Ministries of Education and Health, School of Basic Medical Science and Institutes of Biomedical Sciences, Shanghai Medical College of Fudan University, Shanghai 200032, China；

* Corresponding authors: Di Qu

Tel: 86-21-54237524(D. Qu)

Fax: 86-21-64227201 (D. Qu),

E-mail addresses: dqu@fudan.edu.cn (D. Qu)

[†] These authors contributed equally to this work.

Table S1. The derivatives combined with vancomycin or cefazolin against *S. epidermidis* ATCC 35984

**Derivatives**	**Antibiotics**	**MIC (mg/L)**			**FICI**[a]	**Synergy**[b]
		Derivative alone	Antibiotic alone	In combination (Derivative/Antibiotic)		
H2-38	van[c]	0.8	4	0.4/0.5	0.63	-
	cefaz[d]	0.8	8	0.4/0.5	0.56	-
H2-39	van	0.8	4	0.8/0.5	1.13	-
	cefaz	0.8	8	0.4/0.25	0.53	-
H2-57	van	1.7	4	1.7/0.5	1.13	-
	cefaz	1.7	8	0.85/0.25	0.53	-
H2-60	van	1.7	4	0.85/0.25	0.56	-
	cefaz	1.7	8	0.85/0.25	0.53	-
H2-74	**van**	**0.8**	**4**	**0.2/0.5**	**0.38**	+
	cefaz	0.8	8	0.4/1	0.63	-
H2-81	**van**	**0.7**	**4**	**0.18/1**	**0.5**	+
	**cefaz**	**0.7**	**8**	**0.18/1**	**0.38**	+

[a] The FICI was calculated as FIC indexes= ($MIC_{derivative}$ A in combination/$MIC_{derivative}$ A alone)+($MIC_{antibiotic}$ B in combination/$MIC_{antibiotic}$ B alone).

[b] The interaction between the two antibacterials is noted as follows: FIC index≤0.5, Synergy (+); 0.5<FIC index≤4 (−), No interaction; FIC≥4.0, Antagonism.

[c] van as vancomycin

[d] cefaz as cefazolin
